# Habitat Mapping of *Bos gaurus* in Parsa National Park, Nepal: Ensemble Modeling Approach

**DOI:** 10.1002/ece3.71148

**Published:** 2025-03-20

**Authors:** Anish Dhakal, Dinesh Neupane, Sunjeep Pun, Sheila Ghimire, Manoj Kumar Sah, Bikash Adhikari, Jeetendra Gautam

**Affiliations:** ^1^ Faculty of Forestry Agriculture and Forestry University Hetauda Nepal; ^2^ Zoological Society of London (ZSL) Kathmandu Nepal; ^3^ Resources Himalaya Foundation (RHF) Lalitpur Nepal; ^4^ Department of National Parks and Wildlife Conservation Kathmandu Nepal

## Abstract

*Bos gaurus* , a globally vulnerable and protected priority species in Nepal, has experienced habitat loss and fragmentation, poaching, and diseases. Consequently, their population is isolated significantly in Parsa National Park and Chitwan National Park in Nepal. However, their distribution even in these protected areas is limited to topographical features. This study focuses on habitat suitability modeling of *B. gaurus* in Parsa National Park (PNP) utilizing the ensemble modeling approach to identify key ecogeographical and climatic variables influencing *B. gaurus* suitable habitat and to estimate suitability in and around Parsa National Park, Nepal. After the multicollinearity test, potential ecogeographical variables were integrated with ground presence points for ensemble modeling. The model revealed that distance from waterholes and settlements, slope, and bioclimatic variables highly influenced highly in *B. gaurus* 's habitat suitability. The model found only 31.29% (285.55 km^2^) area as a suitable area for *B. gaurus* distribution in and around PNP. The eastern part of the park (newly extended area around Halkhoriya Lake) and the south‐central section of park show the suitable habitat for *B. gaurus* . However, wildlife‐friendly infrastructure in the East–West Highway (that fragments the park) within park can facilitate *B. gaurus* 's movement among these crucial habitat patches. Future habitat projections under the SSP1‐2.6 climate scenario indicate a gradual reduction in suitable habitat, indicating a marginal impact of the climate change scenario on gaur habitat in the area. These changes highlight the limited vulnerability of *B. gaurus* to climate change and the risk of habitat fragmentation, potentially leading to population declines. However, conservation strategies including maintaining water sources, restoring degraded habitats, particularly in the northern section of the park, and enhancing connectivity through wildlife corridors could ensure long‐term survival.

## Graphical abstract

This study employs ensemble modeling to evaluate habitat suitability for the globally vulnerable Gaur ( *Bos gaurus* ) in and around Parsa National Park, Nepal. The distance from waterholes and settlements, slope, and bioclimatic variables influenced highly in *B. gaurus* 's habitat suitability, resulting in only 31.29% of the area classified as suitable habitat. In the context of climate change, there would be a marginal loss of habitat in the SSP1‐2.6 climate scenario. The findings highlight the necessity for wildlife‐friendly infrastructure to connect fragmented habitat patches and the restoration of water sources within the park.

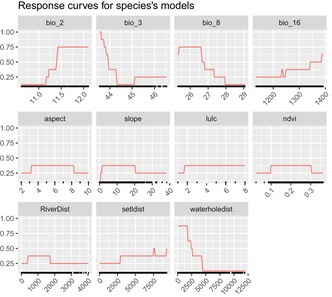

## 1 Introduction

The gaur, the largest wild cattle species in the Bovidae family, weighs between 500 and 1000 kg and is noted for its white “stockings” from knees to hooves and a prominent ridge between its horns, creating a distinctive hollow in its head profile (Ahrestani and Karanth 2014). There are three subspecies: *Bos gaurus* , *B. gaurus readei*, and *B. gaurus* *hubbacki*, found across South and Southeast Asia (Ashokkumar et al. 2011; Duckworth et al. 2016). Historically, gaur occurred in south and southeast Asia, including Sri Lanka. Today, gaur populations are fragmented in Lao PDR, Myanmar, China, Malaysia, India, and Nepal while regionally extinct in Sri Lanka. Over the past century, their global distribution has decreased by 80%, and their numbers have declined by 30% in the last three generations due to hunting, habitat loss, and zoonotic diseases (Duckworth et al. 2016). Less than 20% of their habitat is well‐protected, and the *B. gaurus* subspecies is likely extinct in Bangladesh, with remaining populations in India, Bhutan, and Nepal (Duckworth et al. 2016).

**FIGURE 1:**
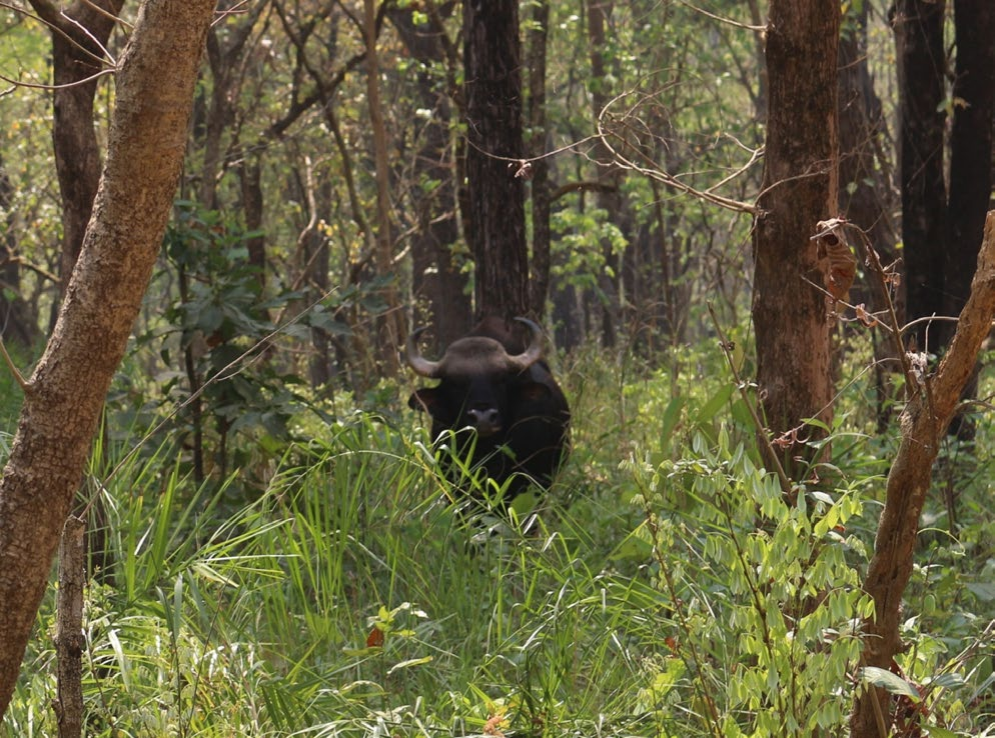
Photo of gaur ( *Bos gaurus* ) spotted in Parsa National Park, Nepal during the gaur count survey 2022. *Source:* Gaur Count Team—PNP 2022.

Gaur inhabits elevations up to 2800 m above sea level and is known for its adaptability as a browser and grazer (Chetri 2006; Gad and Shyama 2009). Overall, individual males typically have a home range of 135–142 km^2^, while individual female home ranges vary from 32 to 169 km^2^, influenced by seasonal vegetation changes, group size, water availability, and rutting behavior (Sankar et al. 2013; Ahrestani and Karanth 2014). In Nepal, the *B. gaurus* , also known as “Gauri Gai,” in the local language (Figure 1). The species is listed in appendix I of CITES (2024), categorized as Vulnerable on the IUCN Red List (VU), and protected under Schedule 1 of the National Parks and Wildlife Conservation Act (1973). Historically, this species inhabited the entire Terai and Siwalik regions, but land use changes, development activities, poaching, and deforestation have isolated *B. gaurus* populations significantly in PNP and Chitwan National Park (CNP) (DNPWC 2020).

Wildlife habitat suitability pertains to understanding how well a specific area represents the probability of occurrence of the species and is impacted by resources and environmental variables (Hirzel and Le Lay 2008). Habitat suitability is dynamic and is crucial for the conservation and management of the species. Habitat suitability assessments comprise three processes: performing field observations for data collection, ecological modeling to identify potentially suitable areas, and expert judgment for verification. This approach has been widely used in various aspects of conservation, including habitat preference for larger herbivores (Neupane et al. 2019, 2020; Pant et al. 2021; Pun et al. 2022), connectivity (Neupane et al. 2022), and climatic scenario projection (Baral et al. 2023; Dhami et al. 2023) in Nepal. Recent advancements in machine learning and computational tools have enhanced the precision of habitat suitability modeling (Ghareghan et al. 2020) providing valuable insights for developing effective conservation and management strategies.

Globally, several studies have explored the habitat preferences and distribution of *B. gaurus* in various landscapes. For instance, studies in India have focused on the gaur's habitat use in tiger reserves such as Bandhavgarh (Sankar et al. 2013) and Tadoba‐Andhari (Paliwal and Mathur 2012), highlighting the importance of water sources, grasslands, and forest cover for gaur survival. Similarly, research in Malaysia has examined the connectivity of gaur habitats in fragmented landscapes, emphasizing the need for corridors to maintain genetic diversity and population viability (Ariffin et al. 2021). In Bhutan, studies have documented gaur distribution and abundance in Royal Manas National Park, underscoring the species' preference for lowland forests and riverine habitats (Zangmo et al. 2018).

In Nepal, research on *B. gaurus* has been limited, with most studies focusing on population counts and general habitat descriptions rather than detailed habitat suitability modeling. For example, Chetri (2006) conducted a diet analysis of gaur in Parsa Wildlife Reserve, while Lamichhane et al. (2018) documented the rapid recovery of tiger populations in Parsa, indirectly highlighting the importance of prey species such as gaur. Poudel et al. (2024) studied the summer season habitat use of gaur in Chitwan National Park and identified that the canopy cover and forest and road distance increase the probability of gaur occurrences. However, there remains a significant gap in understanding the specific ecological and climatic factors influencing gaur habitat suitability in Nepal, particularly in the context of climate change and habitat fragmentation.

Identifying these habitat needs, including both biotic and abiotic environmental variables, is essential for effective conservation (Cañadas et al. 2005). Protecting this species within tiger‐bearing landscapes not only aids in the conservation of *B. gaurus* but also enhances prey–predator dynamics. By examining the ecogeographical variables that influence *B. gaurus* distribution, we can gain valuable insights into managing the species within predator‐dominated landscapes. Larger prey species are more beneficial for tiger populations, and in landscapes with a limited number of large prey, promoting the habitat of such species could help support the growing tiger population (DNPWC and DFSC 2022). To understand the gaur distribution and potential habitats, we applied ensemble modeling to analyze *B. gaurus* distribution using species occurrence data, environmental variables, and spatial attributes (Thuiller et al. 2024), identifying priority areas for future conservation efforts within the park.

Unlike earlier studies that broadly documented *B. gaurus* distribution, our research quantifies the relative importance of ecological and climatic factors, offering a more nuanced perspective on habitat selection. Additionally, this study delves into the traditional view that *B. gaurus* primarily inhabits dense forests by demonstrating its preference for mixed landscapes with access to water. By incorporating ensemble modeling, we improve predictive accuracy, setting a new standard for habitat suitability assessments in conservation planning. Furthermore, our climate projections provide the first quantitative analysis of how future environmental changes may impact *B. gaurus* habitat, underscoring the need for adaptive management strategies. These contributions are crucial for developing targeted conservation interventions, ensuring the long‐term survival of *B. gaurus* in PNP and beyond.

## 2 Materials and Methods

### 2.1 Study Area

**FIGURE 2:**
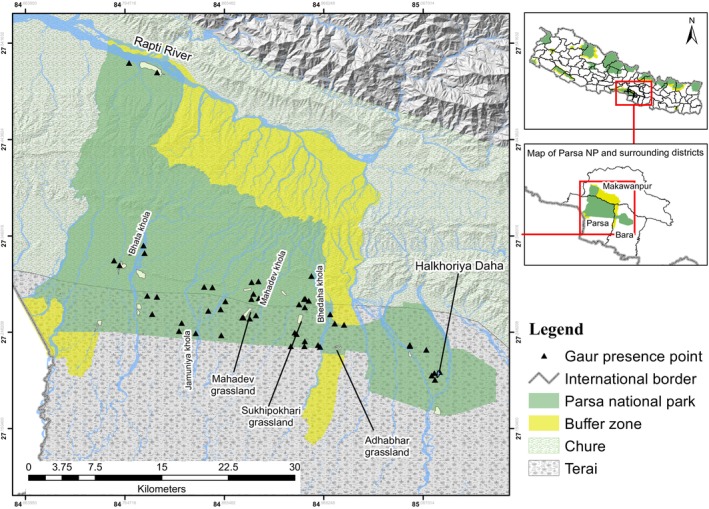
Important habitat features and current gaur presence locations in Parsa National Park and its buffer zone, Nepal.

Parsa National Park (area: 627.39 km^2^ with a buffer zone of 285.3 km^2^) is situated in central Nepal, spanning the Madesh and Bagmati Provinces. The park is located between latitudes 27°15′ N and 27°33′ N and longitudes 84°41′ E and 84°58′ E. PNP extended its area by 128.39 km^2^ in 2015 to reach its current size to fulfill the lack of wet areas for herbivores. However, the extended area is fragmented from the western part of the park by the east–west highway. It is linked with CNP in the west, connected with Valmiki Tiger Reserve, India, in the west‐southern part, and with the national forest in the north and west direction (PNP 2018) (Figure 2). The park features artificially constructed water holes, natural lakes, and approximately 5.3 km^2^ of grasslands, supporting a diverse range of wildlife including 37 mammal species, 490 bird species, and 336 plant species (PNP 2018). The Terai and Siwalik areas are predominantly covered by Sal forest (90%), mixed hardwood, and riverine forests of Sissoo and Khair, with smaller grassland areas (PNP 2018). National Park can generally be categorized into three topographic zones running from north to south: the Churia (Siwalik) range, the Bhawar region, and the Terai (PNP 2018). The Chure and Siwalik zones (lying above 250 msL) jointly cover about two‐thirds (62.5%) area of the park while Terai and inner‐Terai occupy the remaining part (DNPWC 2020). PNP experiences a humid subtropical climate with four distinct seasons—summer, monsoon, winter, and spring. During summer (May–July), temperatures can reach up to 39°C, making the area extremely hot and dry, but in the monsoon season (July–September), rainfall brings slight relief with a modest decrease in temperature, while winter, from November to January, is marked by cold mornings and evenings, with temperatures dropping to as low as 7°C under clear skies (PNP 2018). Numerous seasonal streams flow through the park in the Chure watershed systems, but a few perennial rivers also exist (Rapti Khola along the Northern boundary and Bhata Khola in the South direction) in the area. Along with this, there is Halkhoria Lake in the extended area (eastern part). All other streams such as Pasaha, Dudhoura, Bhedaha, Bhalu, Mahadev, and Jamuniya are replenished in the monsoon (June–September) and dried in winter (November–January) (PNP 2018) (Figure 2).

## 3 Methods

### 3.1 Preliminary Survey

Field visits and observations, along with informal discussions with park authorities, the Nepal Army, the Zoological Society of London (ZSL) field office, and park staff, were conducted to gain an overview of the study area and vegetation composition. Additionally, frequent *B. gaurus* observation sites, identified from the park's patrolling data, were visited for data collection. Secondary data from the *B. gaurus* count survey was also utilized for reference.

### 3.2 Habitat Survey

The area was divided into 2 × 2 km grids to facilitate detailed observation of *B. gaurus* (Paliwal and Mathur 2012; Zangmo et al. 2018) using the “grid index feature” tool in ArcGIS 10.8 software. Out of 221 grids, 97 were selected for the survey based on preliminary field visits, daily patrolling data, input from park staff, and accessibility of areas. Within each grid, a transect survey of 2 km was conducted along existing paths and fire lines in PNP. The only direct sightings of *B. gaurus* were recorded and used for analysis. The survey was carried out between February and May 2022. We used only 52 presence records (only direct sightings) from the field surveys, *B. gaurus* count survey, and data provided by ZSL Parsa for habitat suitability modeling within Parsa National Park.

### 3.3 Environmental Variables

A total of 27 variables, including eight ecogeographical and 19 bioclimatic variables (Table S1), were preliminarily selected based on the literature on *B. gaurus* habitat requirements for this study (Ahrestani and Karanth 2014; Ashokkumar et al. 2011; Imam and Kushwaha 2013; Paliwal and Mathur 2012; Zangmo et al. 2018; Ariffin et al. 2021). The slope and aspect were created from DEM (Digital Elevation Model) in ArcGIS 10.8 with a spatial analyst tool, where aspect was categorized into 10 classes (Table S1). All layers, including ecogeographical and bioclimatic variables, were cropped by PNP boundary and then converted to raster TIFF format (Tagged Image File Format) with spatial analyst tools, and then resampled to a 30 m cell size with a resample tool and converted to UTM 45 N, WGS 84 projection system with project raster tool in ArcGIS 10.8. A database on waterholes was created during fieldwork to update the park's geospatial database. Then, the Variance Inflation Factor (VIF) was calculated to detect multicollinearity in a regression analysis using the “usdm” package in R‐Studio (Naimi 2023). A total of 11 variables with VIF values below 10 (following Neupane et al. 2017) were selected for habitat suitability modeling (Table S2).

### 3.4 Current Habitat Suitability Modeling Using the Ensemble Modeling Approach

Since ensemble modeling can integrate multiple models and improve the accuracy, reliability, and robustness of predictions (Araújo and New 2007; Marmion et al. 2009) we used it for habitat suitability modeling of gaur using the ‘biomod2’ package in R (Thuiller et al. 2024) with cross‐validation modeling parameter. We selected 10 algorithms: Artificial Neural Network (ANN), Classification Tree Analysis (CTA), Flexible Discriminant Analysis (FDA), Generalized Additive Model (GAM), Generalized Boosting Model (GBM) or Boosted Regression Trees (BRT), Generalized Linear Model (GLM), Multiple Adaptive Regression Splines (MARS), Maximum Entropy (MAXENT), Random Forest (RF), and Surface Range Envelope (SRE) or BIOCLIM to create the ensemble model. We found no trends or outliers in the presence points; however, Random Forest would help to handle them relatively well. Usually, in *k*‐fold cross‐validation, the dataset is divided into *k* subsets (folds) while the model is trained on *k* − 1 folds and validated on the remaining fold, and this process is repeated *k* times, and the results are averaged to provide a more robust evaluation (James et al. 2013). We generated 1000 pseudo‐absence points using the “sre” selection method. The presence and pseudo‐absence points were divided into two datasets: 70% for training and 30% for testing to evaluate the model. This process resulted in 70 model runs, three pseudo‐absence selection runs, and three evaluation runs across the 10 methods. In this study, we calculated Area Under Curve (AUC) and True Skill Statistics (TSS) to assess the predictive performance of the ensemble model. AUC measures the trade‐off between sensitivity (true positive rate) and specificity (true negative rate), making it ideal for presence–absence modeling, whereas TSS accounts for true positive and negative rates, emphasizing reliability in prediction (Allouche et al. 2006; Araújo and New 2007). AUC values closer to 1 indicate excellent model performance, while TSS values above 0.6 generally indicate strong and reliable predictive power (Allouche et al. 2006). Models with TSS values below 0.6, specifically ANN and SRE, were excluded, while the remaining eight models (CTA, FDA, GAM, GBM, GLM, MARS, MAXENT, RF) with TSS values greater than 0.6 (Allouche et al. 2006; Marmion et al. 2009) were selected to construct the ensemble model using a weighted mean strategy (Table S3). Additionally, response curves were generated to understand the effects of environmental variables on ensemble predictions. The resulting TIFF raster image of the model was further analyzed using ArcGIS software version 10.8 and classified into four suitability classes based on the equal interval reclassify method: unsuitable area (0–0.25 value), low‐medium (0.25–0.5 value), medium to high (0.5–0.75 value) and optimum (0.75–1 value) for *B. gaurus* distribution.

### 3.5 Future Habitat Suitability Using Ensemble Modeling Approach

We utilized future bioclimatic data from the MIROC6 model, part of the Models for Interdisciplinary Research on Climate (MIROC), to assess how *B. gaurus* might respond to future climate conditions. MIROC6 is the suitable global circulation model (GCM) in predicting future climate over the geographical range of *B. gaurus* (Mishra et al. 2014; Pant et al. 2021). We selected bioclimatic variables representing greenhouse gas (GHG) concentration pathways aligned with Shared Socioeconomic Pathways (SSPs): SSP1‐2.6 for two future timeframes, 2050 and 2070. For this study, we assumed that topographic variables would remain unchanged over time.

## 4 Result

### 4.1 Environmental Variables for *B. gaurus* Presence

**FIGURE 3:**
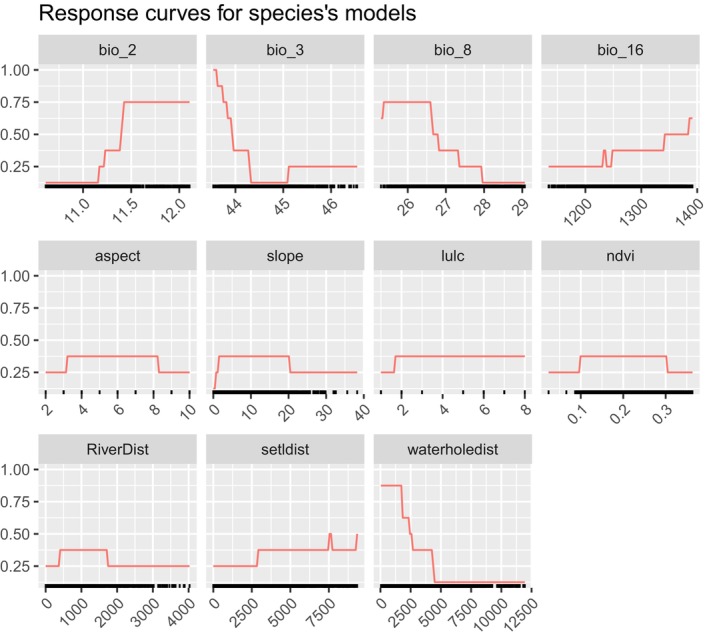
Response curves for the predicted variables to model gaur habitat suitability in and around Parsa National Park, Nepal. The response curves were derived from the model using bioclimatic and environmental variables. The *y*‐axis represents habitat suitability values ranging from 0 to 1, while the *x*‐axis shows the range of values for each variable.

The four bioclimatic variables: mean diurnal range of temperature (bio_2), isothermality (bio_3), mean temperature of the wettest quarter (bio_8), and precipitation of the wettest quarter (bio_16) and seven ecogeographical variables: distance from the waterhole, settlement, distance from river, slope, aspect, ndvi, and lulc showed some influences on habitat suitability modeling of *B. gaurus* in and around Parsa National Park. Distance from the waterhole with a mean importance score of 0.405 was the most influential variable (28.14% contribution to models) followed by mean diurnal range (18.07%), isothermality (14.66%), settlements distance (14.11%), slope (8.90%) and mean temperature of the wettest quarter (7.92%) (Table 1) in habitat suitability study in and around Parsa National Park. The study found that *B. gaurus* shows a positive response towards the area with the southeast and south aspects, slope less than 8°, land cover (water sources, forest, grassland, flood vegetation, and bare ground), ndvi values of 0.22–0.32, within river distance of 1200 m, beyond 6000 m from human settlement, and within 1500 m from the waterhole (Figure 3).

**TABLE 1:** Average variable importance of predicted variables used for the ensemble of the small models for *gaur* presence in and around Parsa National Park, Nepal.

Variable	Mean variable importance	Percentage
Waterhole distance (waterholedist)	0.405	28.14
Mean diurnal range (bio2)	0.26	18.07
Isothermality (bio3)	0.211	14.66
Settlement distance (setldist)	0.203	14.11
Slope	0.128	8.90
Mean temp. wettest quarter (bio8)	0.114	7.92
Precipitation of wettest quarter (bio16)	0.05	3.47
River distance (RiverDist)	0.037	2.57
ndvi	0.02	1.39
Aspect	0.01	0.69
lulc	0.001	0.07

### 4.2 Current Habitat Suitability of *B. gaurus*

**FIGURE 4:**
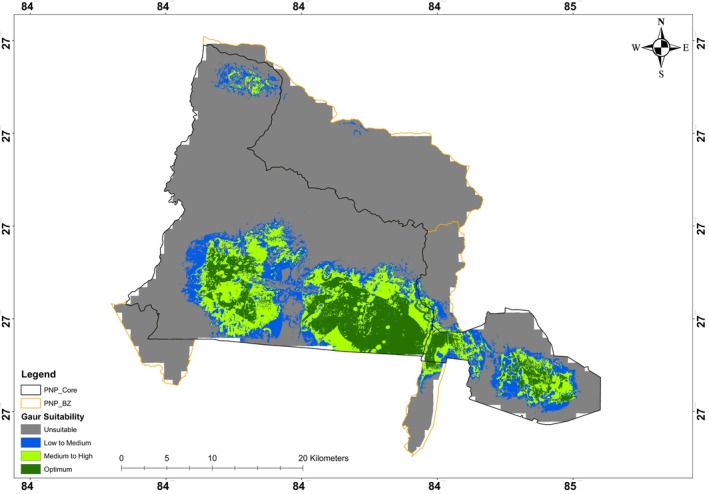
Predicted current suitable habitats for gaur in and around Parsa National Park, Nepal, by the ensemble model after application of the presence/absence threshold.

About 68.71% of the area (627.14 km^2^) of the PNP and buffer zone was predicted to be unsuitable and 31.29% (285.55 km^2^) area was categorized as a suitable (low‐medium: 96.37 km^2^ [10.56%], medium to high: 101.82 km^2^ [11.16%] and optimum: 87.37 km^2^ [9.57%]) for *B. gaurus* distribution (Table 2 and Figure 4). Despite their minimal presence in the park's extension area, most of the extension area was identified as a suitable habitat for the *B. gaurus* . Most of the area, particularly the park's northern part in the Chure hills, was found unsuitable for *B. gaurus* .

**TABLE 2:** Suitable area predicted by an ensemble of small models for gaur in current and future changes in and around Parsa National Park, Nepal.

Climate scenario	Unsuitable habitat in km^2^ (%)	Suitable habitat in km^2^			
		Low to medium (%)	Medium to high (%)	Optimum	Total suitable habitat (%)
Current	627.14 (68.71)	96.37 (10.56)	101.82 (11.16)	87.37 (9.57)	285.55 (31.29)
SSP1‐2.6 (2041–2060)	633.61 (Gain by 1.03)	91.30 (Loss by 5.26)	73.20 (Loss by 28.11)	114.59 (Gain by 31.15)	279.08 (Loss by 2.27)
SSP1‐2.6 (2061–2080)	635.13 (Gain by 1.27)	96.88 (Loss by 0.53)	69.71 (Loss by 31.54)	110.97 (Gain by 27.01)	277.56 (Loss by 2.80)

*Note:* SSP is shared socioeconomic pathways as obtained from the Models for Interdisciplinary Research on Climate (MIROC).

### 4.3 Future Habitat Suitability for *B. gaurus*

**FIGURE 5:**
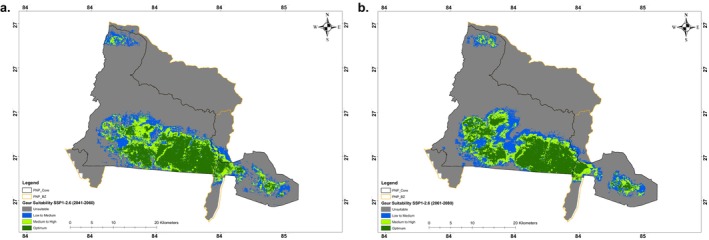
Projection of gaur habitat suitability under modeled shared socioeconomic pathways (SSP1‐2.6) for the period (a) 2041–2060 and (b) 2061–2080 as predicted by the ensemble of small models in and around Parsa National Park, Nepal.

The period 2041–2060 revealed that the unsuitable habitat area increases slightly to 633.61 km^2^, marking a gain of 6.47 km^2^ (1.03%) compared to the current scenario (Table 2 and Figures 4 and 5a). Specifically, the low‐to‐medium habitat suitability decreases by 5.07 km^2^ (5.26%), while medium‐to‐high suitability experiences a significant loss of 28.62 km^2^ (28.11%). Additionally, for the period 2061–2080, the total suitable habitat area continues to decline slightly, reaching 277.56 km^2^, which represents a further loss of 7.99 km^2^ (2.80%) (Table 2 and Figures 4 and 5b).

## 5 Discussion

### 5.1 Factors Influencing the Habitat Selection of *B. gaurus* in PNP

The response curves generated through the Ensemble model shed light on the complex interactions between *B. gaurus* and the ecological and bioclimatic factors influencing its habitat suitability. The model's overall AUC value of 0.974 and TSS value of 0.798 indicate its strong predictive power, and the insights obtained from the data are critical for informing conservation strategies. *Bos gaurus* prefers to inhabit areas near water sources from rivers (river floodplains) and waterholes. Water sources not only serve as drinking locations but also support the growth of lush vegetation, essential for grazing. This finding is consistent with previous studies (Poudel et al. 2024; Zangmo et al. 2018; Gad and Shyama 2009; Chetri 2006; Choudhury 2002), which highlight that *B. gaurus* tend to favor areas with abundant food and water, including grasses and the tender leaves of shrubs and bushes. In PNP, the predicted suitable habitat around the Mahadev and Bhedaha area is influenced by the presence of waterholes (such as Sukepokhari waterhole, Mahadev 6 waterhole, and Kalidaha waterhole) and grassland (such as Sukepokhari grassland, and Aadhabar grassland) serves as a significant food source for the species. Having permeable porous sediment in Parsa National Park, over 70% of the park faces water scarcity throughout the dry months (Lamichhane et al. 2018), and the artificial waterholes provide drinking and feeding grounds during dry months. Strategies to maintain water sources, especially during dry seasons, should prioritize the maintenance of natural and artificial waterholes to assure the continuous water supply in the area.

Study identified a higher preference for habitat towards undisturbed areas far from human settlement, indicating that there would be a low probability of human–*gaur* conflict; however, habitat fragmentation would decrease their survival. Being a large herbivore, this species is particularly sensitive to human disturbances, which, if continued, can disrupt foraging patterns and breeding (DNPWC 2020). Thus, minimizing human impacts in *B. gaurus* habitats, particularly through the creation of buffer zones in the southern area of PNP and limiting habitat fragmentation through wildlife‐friendly infrastructure development, should be a management priority to secure their survival in the area.

The species prefer flat or gently sloping terrain (slopes < 10°), which may provide easier access to grazing areas and water while minimizing energy expenditure during foraging. Lack of sloped land aids in habitat choice by this species as it prefers the plain area in comparison to hilly land (Imam and Kushwaha 2013; Sankar et al. 2013; Ahrestani and Karanth 2014). The species also appears to favor Southeast and South aspects, possibly reflecting a preference for specific sunlight exposure and moisture conditions conducive to vegetative growth. Being a dry zone of the Chure hills in the northern part of the park, *B. gaurus* predominantly inhabits the foothills of the Chure region in the southern part, especially in Bhata, Charbhaiya, Mahadev Khola, Bhedaha Khola, and Halkhoria Daha (PNP 2018; DNPWC 2020).

*Bos gaurus* favors moderately vegetated areas, suggesting that the species requires environments with sufficient vegetation to support its grazing needs but may avoid areas with overly dense vegetation that could impede movement or visibility. This fact is supported by previous studies (Paliwal and Mathur 2012; Imam and Kushwaha 2013; Sankar et al. 2013; Zangmo et al. 2018). Furthermore, the response curve for land use and land cover change shows higher suitability in land use categories corresponding to natural habitats such as forests and grasslands, with significantly lower suitability in human‐modified landscapes such as built areas and croplands. This reinforces the need for preserving large, contiguous areas of natural habitat to support healthy populations.

The species appears to favor areas where the mean diurnal range is moderate, around 11°C, and the mean temperature during the wettest quarter is approximately 27°C–280°C. *Bos gaurus* appears to favor regions with moderate temperatures during the wettest quarter. These conditions likely offer optimal thermal comfort and support vegetation growth during the wet season. The physiological tolerance of *B. gaurus* to moderate temperatures aligns with its distribution in tropical and subtropical regions (Duckworth et al. 2016). Additionally, *B. gaurus* prefers regions where there are environments with stable daily temperatures but significant seasonal changes. This adaptability to temperature variation may reflect the species' preference for tropical environments, where temperatures fluctuate seasonally and daily.

Precipitation during the wettest quarter strongly influences *B. gaurus* distribution, with peak suitability at 1300–1350 mm of rainfall. This amount of precipitation ensures adequate water availability and supports the growth of lush vegetation, essential for foraging. Too much or too little rainfall reduces habitat suitability, suggesting that *B. gaurus* requires a consistent balance between water availability and vegetation productivity. With climate change threatening to disrupt rainfall patterns, these results highlight the need for climate‐resilient conservation strategies to anticipate future shifts in habitat suitability.

### 5.2 Importance of Extension Area for *B. gaurus* Population

One‐third of the area in the Parsa National Park and its buffer zone was found suitable for the gaur population, of which the majority lies in the extended area and southern part of the park. The habitat prediction emphasizes the significance of the extension area of PNP, particularly around Halkhoriya Lake. Almost all areas in the extension part are predicted as suitable, indicating a strong preference for this area by the species. It is further supported by the fact that *B. gaurus* favors the habitat that has compact grassy areas adjacent to water resources (Ahrestani and Karanth 2014; Ashokkumar et al. 2011; Chetri 2006; Choudhury 2002). Therefore, an extension of habitat in the case of *B. gaurus* has been beneficial in PNP for its population restoration. Despite this, the extension areas play a vital role in conserving the *B. gaurus* population in the park, where their movement is disrupted by the presence of the east–west highway, leading to potential road collisions in the future. The limited recording of *B. gaurus* in the extension part would be the result of road disturbances for the movement of *B. gaurus* in the area. If facilitated by an underpass or overpass along the east–west highway, the *B. gaurus* population may grow more in an eastern extended area.

### 5.3 Projected Climate Change Impacts on *B. gaurus* Habitat Suitability in Parsa National Park

Climate change is expected to alter habitat suitability for *B. gaurus* in PNP. Our analysis indicates a gradual increase in unsuitable habitat over time, with a loss of 2.27% (6.47 km^2^) of suitable habitat by 2041–2060 and an additional 2.80% (7.99 km^2^) loss by 2061–2080. Habitat loss and fragmentation are already major threats to gaur populations (Ashokkumar et al. 2011; Duckworth et al. 2016; DNPWC 2020), and climate‐induced changes could exacerbate these pressures, leading to population declines or local extinctions in certain regions. If gaur populations are confined to shrinking or fragmented habitats, their genetic diversity and resilience to environmental changes may also decline. Thus, expanding habitat management interventions in the northern Chure region and connecting existing suitable habitats to the northern part of the park can minimize the climate change impact on habitat loss.

The projected habitat loss underscores the vulnerability of *B. gaurus* to climate change and the need for adaptive conservation strategies. The decline in medium‐to‐high suitability areas suggests potential fragmentation of the habitat, which may reduce population connectivity and increase risks of local extinctions. Additionally, since *B. gaurus* prefers areas with moderate temperature and precipitation conditions, any significant shifts in these factors may force the species to move toward new locations, potentially increasing human‐wildlife interactions. To mitigate these impacts, conservation efforts should prioritize maintaining water sources, restoring degraded habitats, and enhancing connectivity through wildlife corridors. Future management should also integrate climate‐resilient strategies, such as habitat enrichment and assisted migration, to ensure the long‐term viability of *B. gaurus* in PNP.

## 6 Conclusion and Conservation Implication

This study utilized ensemble modeling to assess habitat suitability for the *B. gaurus* in and around Parsa National Park, providing valuable information for the development of spatially explicit conservation plans to manage and restore *B. gaurus* habitats, ensuring the long‐term survival of this species in the face of human expansion and environmental changes. Such strategies include the continuous maintenance of water sources, human disturbance reduction, and facilitation of connectivity within the park. It is worth noting that this study provides a valuable baseline assessment of *B. gaurus* habitat suitability in PNP and could also support tiger as an abundant gaur population in Parsa National Park can support the recently recovered tiger population (Lamichhane et al. 2018).

*Bos gaurus* prefers forested areas in proximity to water sources, further from human settlements with bioclimatic preferable conditions. The extension areas, particularly around Halkhoriya Lake and the southern part of the park were identified as crucial for *B. gaurus* conservation. However, barriers like the east–west highway disrupt *B. gaurus* movement among these critical habitat patches, emphasizing the need for wildlife‐friendly infrastructure development. Implementing these measures will be vital for ensuring the long‐term survival of *B. gaurus* populations in PNP, particularly in the face of ongoing environmental changes.

Although this study provides important insights into the suitability of gaur ( *B. gaurus* ) habitat in Parsa National Park and highlights important environmental and climatic factors that influence its distribution, the findings indicate that only a limited area of the park is currently suitable for gaurs, which emphasizes the need for targeted conservation efforts for a national park. In a climate change scenario, gaur may use the currently unsuitable habitat as their habitat in the future if proper habitat management interventions such as creating grassland and waterholes are in place, particularly in the northern Chure area. However, the application of the research findings would need further exploration for all regions and global habitats of this species. Limited research on gaur distribution has forced us to restrict the study to Parsa National Park. Expanding research in identifying gaur distribution in other protected areas can help to explore the potential habitat in the entire country. Additionally, long‐term monitoring is essential to assess changes in habitat suitability over time. The limited data on *B. gaurus* presence in this study has constrained our ability to conduct a more in‐depth analysis. Future research should focus on long‐term monitoring of the suitability of habitat, as well as the relationship between habitat availability and actual habitat use. Such an approach would provide more comprehensive insights into gaur conservation and management, enabling better‐informed strategies for preserving this vulnerable species.

## Author Contributions

**Anish Dhakal:** conceptualization (equal), data curation (equal), formal analysis (lead), writing – original draft (equal), writing – review and editing (equal). **Dinesh Neupane:** conceptualization (equal), data curation (equal), formal analysis (supporting), supervision (lead), validation (equal), writing – original draft (equal), writing – review and editing (equal). **Sunjeep Pun:** data curation (equal), formal analysis (equal), writing – review and editing (equal). **Sheila Ghimire:** data curation (equal), formal analysis (equal), writing – review and editing (equal). **Manoj Kumar Sah:** methodology (equal), resources (equal), writing – review and editing (equal). **Bikash Adhikari:** data curation (equal), methodology (equal), writing – review and editing (equal). **Jeetendra Gautam:** data curation (equal), methodology (equal), writing – review and editing (equal).

## Conflicts of Interest

The authors declare no conflicts of interest.

## Supporting information


Data S1.

Table S1.
Table S2.
Table S3.

## Data Availability

All necessary data files are attached in the system and can be accessed by everyone for academic purposes.
