# Supplementary material for: Habitat Mapping of Bos gaurus in Parsa National Park, Nepal: Ensemble Modeling Approach
Source: Ecol Evol. 2025 Mar 20;15(3):e71148. doi: 10.1002/ece3.71148 (PMC11925645; doi:10.1002/ece3.71148)
Supplement: Supplementary file 2 — Table S1. Table S2. Table S3. [file ECE3-15-e71148-s002.docx]

**Supplementary files**

Table S1: Environmental variables used for modeling the gaur habitat suitability in and around Parsa National Park, Nepal.

| **S. N** | **Name of Environmental Variables** | **Unit** | **Hypothesized relationship** | **Data Type** | **Source** |
| --- | --- | --- | --- | --- | --- |
| 1 | Aspect  (1. Flat (-1), 2. North (0-22.5) 3. Northeast (22.5-67.5) 4. East (67.5-112.5) 5. Southeast (112.5-157.5) 6. South (157.5-202.5) 7. Southwest (202.5 - 247.5) 8. West (247.5 - 292.5) 9. Northwest (292.5 -337.5) 10. North (337.5 - 360)) | degree | prefer certain aspects | Raster | modified from DEM <https://earthexplorer.usgs.gov/> |
| 2 | Slope | degree | linearly decreasing | Raster | modified from DEM <https://earthexplorer.usgs.gov/> |
| 3 | Elevation | masl | linearly decreasing | Raster | <https://earthexplorer.usgs.gov/> |
| 4 | Waterhole Distance | m | linearly decreasing | Vector | PNP & ZSL, Bara (Field Survey) |
| 5 | River Distance (River Floodplain Distance) | m | linearly decreasing | Vector | ICIMOD (Regional Data Base System, Nepal)  ([ICIMOD \| RDS](https://rds.icimod.org/Home/Data?any=Settlement&Category=datasets&&themekey=Nepal&&page=1)) |
| 6 | Normalized Difference Vegetation Index (NDVI) | unitless | linearly increasing | Raster | <https://earthexplorer.usgs.gov/> |
| 7 | Land Use and Land Cover (LULC) (1. Water, 2. Forest, 3. Grassland, 4. Flood vegetation, 5. Cropland, 6. Shrubland, 7. Built area, 8. Bare land) | unitless | prefer certain landcovers | Raster | [ICIMOD \| RDS](https://rds.icimod.org/Home/Data?any=Settlement&Category=datasets&&themekey=Nepal&&page=1) |
| 8 | Settlement Distance | m | linearly increasing | Vector | [ICIMOD \| RDS](https://rds.icimod.org/Home/Data?any=Settlement&Category=datasets&&themekey=Nepal&&page=1) |
| 9 | Bioclimatic Variable (Bio_1 to Bio_19) | ^0^C/%/mm | influenced with particular variables | Raster | <https://www.worldclim.org/data/index.html> |

Table S2: List of environment variables (with their VIF values) used in the final modelling for habitat suitability of gaur in Parsa National Park, Nepal

| **Variables used** | **VIF** |
| --- | --- |
| Mean diurnal range (bio2) | 6.19 |
| Isothermality (bio3) | 4.39 |
| Mean temp. wettest quarter (bio8) | 4.62 |
| Precipitation of wettest quarter (bio16) | 1.44 |
| aspect | 1.05 |
| slope | 1.94 |
| lulc | 1.53 |
| ndvi | 1.29 |
| River Distance (River Floodplain Distance) (RiverDist) | 1.31 |
| Settlement Distance (setldist) | 2.18 |
| Waterhole Distance (waterholedist) | 1.81 |

Table S3: Evaluation Matric of Methods used in ensemble modeling for habitat suitability analysis for gaur in and around Parsa National Park, Nepal. This table summarizes the performance metrics of various habitat suitability models. The statistics include the Area Under the Curve (AUC) and True Skill Statistic (TSS), which assess the predictive accuracy and reliability of each model. The table also lists the R packages utilized for model implementation, highlighting the tools used for each algorithm. The bold models showed TSS below 0.6 thus were excluded from final ensemble model.

| S.N. | Models | Statistics Value (AUC) | Statistics Value (TSS) | R -Package |
| --- | --- | --- | --- | --- |
| **1** | **ANN** | **0.771** | **0.524** | **nnet** |
| 2 | CTA | 0.954 | 0.885 | rpart |
| 3 | FDA | 0.886 | 0.649 | mda |
| 4 | GAM | 0.890 | 0.658 | mgcv |
| 5 | GBM | 0.957 | 0.838 | gbm |
| 6 | GLM | 0.917 | 0.722 | stats |
| 7 | MARS | 0.947 | 0.835 | earth |
| 8 | MAXENT | 0.889 | 0.707 | MAXENT |
| 9 | RF | 1.000 | 1.000 | randomForest |
| **10** | **SRE** | **0.714** | **0.427** | **biomod2** |
| 11 | ENSEMBLE | 0.974 | 0.798 | biomod2 |
